# Evaluation of the FAST-M maternal sepsis intervention in Pakistan: A qualitative exploratory study

**DOI:** 10.1371/journal.pone.0284530

**Published:** 2023-04-24

**Authors:** Sheikh Irfan Ahmed, Bakhtawar M. Hanif Khowaja, Rubina Barolia, Raheel Sikandar, Ghulam Kubra Rind, Arshia Jahangir, Fahmida Parveen, James Cheshire, Catherine Dunlop, Pammla Margaret Petrucka, Lumaan Sheikh, Arri Coomarasamy, David Lissauer

**Affiliations:** 1 Aga Khan University Hospital, Karachi City, Pakistan; 2 LUMHS Hospital, Liaquat University of Health and Medical Sciences, Hyderabad City, Pakistan; 3 Aga Khan University, Karachi City, Pakistan; 4 Institute of Metabolism and Systems Research, University of Birmingham, Birmingham, United Kingdom; 5 University of Saskatchewan, Saskatoon, SK, Canada; 6 Institute of Life Course and Medical Sciences, University of Liverpool, Liverpool, United Kingdom; Aga Khan University pakistan, PAKISTAN

## Abstract

**Introduction:**

The World Health Organization and partners developed and evaluated a maternity-specific sepsis care bundle called ‘FAST-M’ for low-resource settings. However, this bundle has not yet been studied in Asia. Our study sought to evaluate the perceptions of healthcare providers about the implementation of the FAST-M intervention in Pakistan.

**Materials and methods:**

The study was conducted at a public sector hospital in Hyderabad. We conducted three focus group discussions with healthcare providers including doctors, nurses, and healthcare administrators (n = 22) who implemented the FAST-M intervention. The Consolidated Framework for Implementation Research was used as a guiding framework for data collection and analysis. The data were analyzed using a thematic analysis approach and deductive methods.

**Results:**

Five overarching themes emerged: (I) *FAST-M intervention and its significance* including HCPs believing in the advantages of using the intervention to improve clinical practices; (II) *Influence of outer and inner settings* including non-availability of resources in the facility for sepsis care; (III) *HCPs perceptions about sustainability*, which were positive (IV) *Integration into the clinical setting* including HCPs views on the existing gaps, for example, shortage of HCPs and communication gaps, and their recommendations to improve these; and (V) *Outcomes of the intervention* including improved clinical processes and outcomes using the FAST-M intervention. Significant improvement in patient monitoring and FAST-M bundle completion within an hour of diagnosis of sepsis was reported by the HCPs.

**Conclusions:**

The healthcare providers’ views were positive about the intervention, its outcomes, and long-term sustainability. The qualitative data provided findings on the acceptability of the overall implementation processes to support subsequent scaling up of the intervention.

## Introduction

Maternal sepsis is the third leading cause of preventable pregnancy-related deaths worldwide [1]. It is a life-threatening condition defined as organ dysfunction resulting from infection during pregnancy, childbirth, post-abortion, or postpartum period [2]. Undetected or poorly managed maternal infections can lead to maternal sepsis, death, disability, and other adverse outcomes [3]. The World Health Organization (WHO) estimates that the global prevalence of maternal sepsis from 2010 to 2016 was 4.4% [4]. The incidence of maternal sepsis in high-income countries is 9–49/ 100 000 deliveries [4], however, in low- and middle-income countries (LMIC), the incidence of maternal sepsis is unidentified due to underreported cases [4]. Despite advancements in healthcare service delivery in the last century, maternal sepsis remains a significant contributor to maternal mortality [2].

Reducing maternal mortality is a priority action in achieving the Sustainable Development Goals (SDGs) through 2030 [5] and gained attention in the United Nations Global Strategy for Women’s, Children’s, and Adolescents’ Health in 2015 [6]. Yet, the efforts and strategies to address Sustainable Development Goal 3.1 of *reducing the global maternal mortality ratio to less than 70 per 100 000 live births* [5] have disproportionally focused on the top two maternal mortality issues, i.e., hemorrhage and hypertensive disorders [6]. Despite maternal sepsis being the 3^rd^ most common cause and a contributing factor in half of the maternal deaths, it has received far less attention [7].

The high maternal mortality rate from maternal sepsis is related to delays in the detection of sepsis cases and their timely management [8]. The prevention, early identification, and adequate management of maternal sepsis will decrease the maternal mortality burden [8]. The burden of maternal mortality from sepsis predominantly falls on LMICs [7]. The World Health Assembly (WHA) adopted a resolution in 2017 to improve the prevention, diagnosis, and management of sepsis [9]. In response, the WHO and other partners launched the ‘Global Maternal and Neonatal Sepsis Initiative’ with the objective to develop and test effective strategies to prevent, detect and successfully manage maternal sepsis [10].

Following this initiative, an evidence-based modified Delphi process was conducted to develop the first maternity-specific sepsis care bundle for use in low-resource settings [11]. The final elements included in the bundle were fluids, antibiotics, source identification and control, transport, and monitoring of the mother and the newborn [11], abbreviated as **FAST-M** for ease of healthcare providers’ (HCPs) recall. The FAST-M intervention was implemented in Malawi and improved the recognition and management of maternal sepsis patients [12]. The FAST-M intervention included i) the modified early obstetric warning score (MEOWS) chart and FAST-M decision tool for identifying maternal sepsis cases, ii) the FAST-M treatment bundle to manage and treat maternal sepsis, (S1 File) and iii) the FAST-M implementation program to train HCPs on using the FAST-M tools and integrate the bundled approach into daily practice [12].

The maternal mortality rate (MMR) in Pakistan was reported as 186 deaths/100,000 live births in 2020 [13]. National figures show that maternal sepsis is the third leading cause of maternal mortality [14]. There are national sepsis guidelines for Pakistan (SGP) that are designed to aid in the identification and management of sepsis in adults in the local settings and are modeled on the Surviving Sepsis Campaign (SSC) [15]. However, these guidelines lack a comprehensive implementation approach for pregnant or recently pregnant populations.

Hence a feasibility study was conducted to assess whether it is feasible to implement the FAST-M intervention for maternal sepsis care in Pakistan [16]. This paper presents the qualitative part of the FAST-M evaluation in Pakistan.

## Materials and methods

### Study design

A two-phase mixed method intervention design was used. This two phase-design began with the adaptation phase. In phase 1 (adaptation), the FAST-M intervention was modified according to the local context before implementation in the study setting. The HCPs provided views on modifications in the bundle care tools according to their local context and a baseline facility survey was conducted to assess the availability of the facility’s resources. The findings of phase 1 have been published as a separate record [17].

Based on the findings of phase 1, two-day training sessions were arranged and delivered by health care experts before the implementation of the FAST-M bundle intervention. The training session provided classroom-based instructions on the FAST-M intervention followed by interactive sessions used as a strategy for hands-on training of healthcare providers on the FAST-M tools. Health care practitioners including doctors and nurses (n = 40) working in obstetrics and gynecology units, internal medicine, operating rooms, emergency, and intensive care units of LUMHS hospital were trained to record patients’ vital signs on the MEOWS chart. Clinicians were also trained on using the FAST-M decision tool, and the health experts provided training on the appropriate administration and the use of treatment bundle elements.

The local clinical champions and team leaders were also identified during training programs and were trained to take the lead in implementation of the FAST-M intervention at the study site and remained engaged throughout the implementation process. These champions were selected from different departments primarily involved in the management of maternal sepsis patients. The selected champions included doctors. The overarching goal of each champion was to encourage engagement and compliance with the FAST-M bundle.

In phase 2, the feasibility assessment of the adapted FAST-M intervention was conducted through quantitative assessments for before-after implementation outcomes (the findings will be detailed elsewhere).

**Fig 1:**
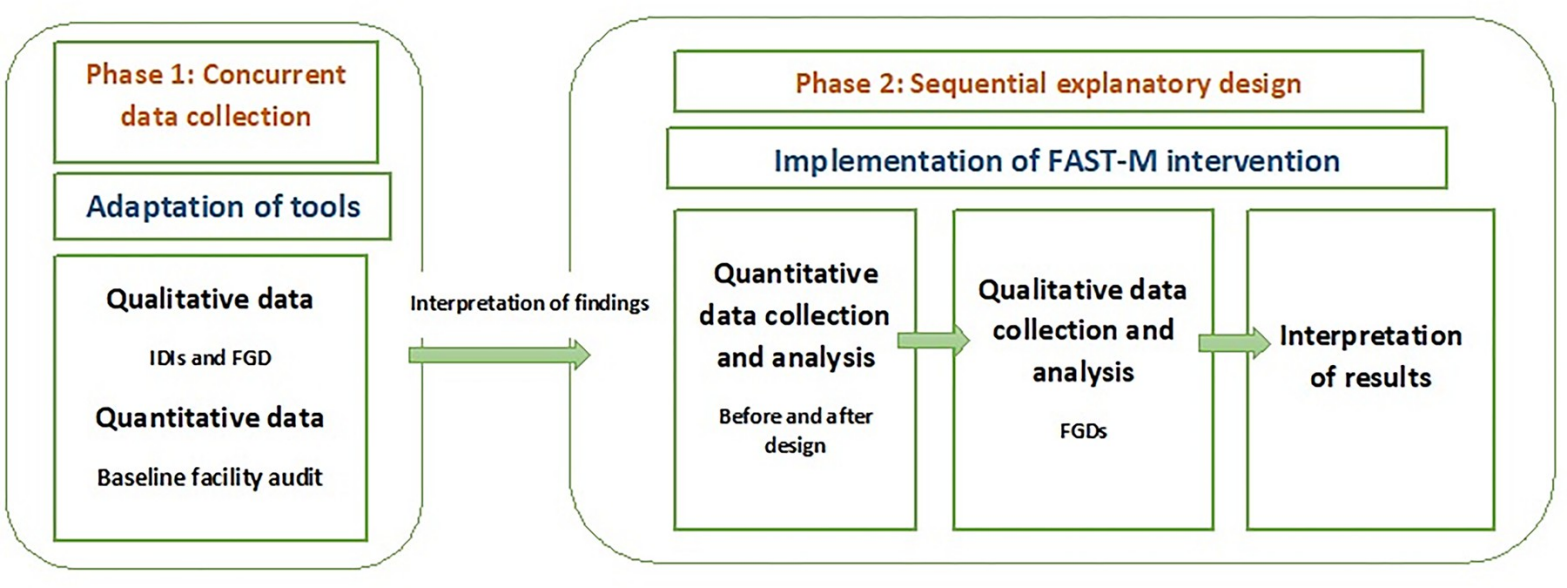
Flowchart displaying study processes.

While the quantitative findings (in phase 2) reflected an improvement in early detection and management of maternal sepsis cases through the use of intervention, these could not explain ‘*what’* the HCPs experiences were in implementing this innovative approach in their health setting, or ‘*how well’* this intervention was integrated into their existing setup. We, therefore, conducted a qualitative study of HCPs and health administrators views and experiences post-implementation, with the objectives of understanding how useful they found this intervention for improving patients and clinical outcomes and what their views were on the overall implementation process. We conducted focus group discussions (FGDs) with HCPs and administrative individuals who were involved in the implementation of the FAST-M intervention. Fig 1 provides the summary of the study processes.

**Fig 2:**
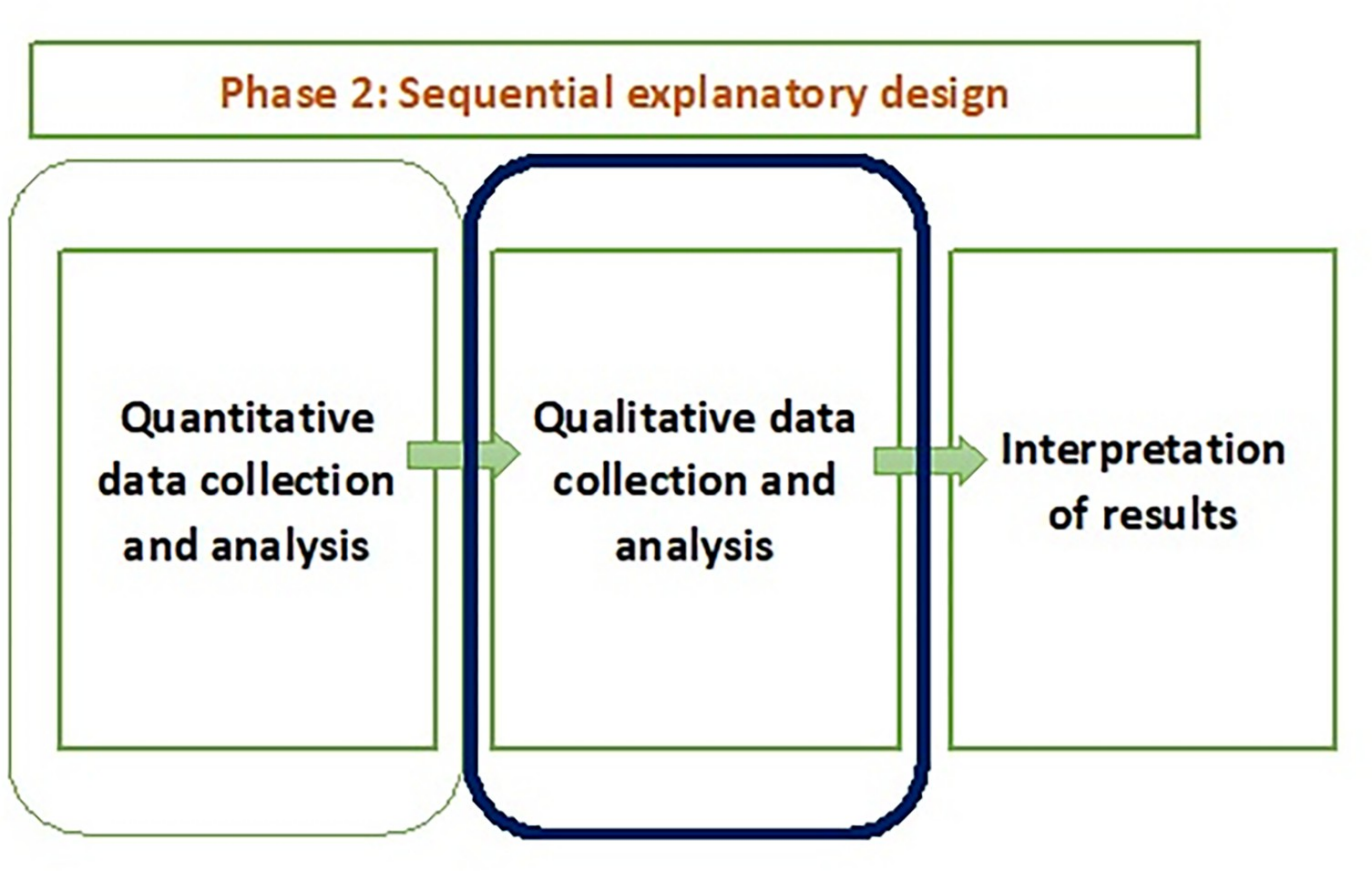
Phase 2 of the FAST-M intervention study.

Phase 2 determined the feasibility of the adapted FAST-M intervention in a low-resource setting in Pakistan through before and after implementation outcomes (QUAN). For the latter portion of the study (Phase 2), we used a qualitative approach [18] (Fig 2) to elicit healthcare professionals experiences, thoughts, and opinions on implementation outcomes such as its usefulness, and long-term sustainability of the intervention (QUAL). The exploration of their perceptions throughout this qualitative work (Fig 2) sought to help us to better understand the individuals’ experiences of the intervention.

### Study setting

Liaquat University of Medical Health Sciences (LUMHS) is in the Hyderabad district of the province of Sindh, Pakistan. LUMHS is a 1300 bed tertiary referral public sector hospital that serves a large number of primarily underprivileged populations and the hospital’s funding has been allocated from the provincial sector government budget.

The hospital offers both in-patient and out-patient services including three Obstetrics and Gynecology (OBGYN) units and the provision of 24 hours emergency coverage to patients coming from urban and rural areas of Sindh. It manages a high volume of cases of maternal sepsis every month.

The facility data shows that the case facility rate of sepsis in pregnant and recently pregnancy women was high 80/370 (21.6%) from January to December 2021 affirming the need for a robust system for early detection and management of maternal sepsis cases in the hospital.

The HCPs from different units and departments, such as the emergency, OBGYN, internal medicine, and intensive care units, were involved from the beginning of this study to plan and design the conduct of the study. These departments primarily deal with maternal sepsis patients, therefore we decided to have views of providers from all the units and not to limit it to only OBGYN. The FAST-M intervention was implemented in all these specified units to compare the before and after outcomes in clinical care and patients’ outcomes.

### Ethics approval

Ethical approval for this study was obtained from the LUMHS hospital [REC/-886, 4–87], Aga Khan University Ethical Review Committee [2019-2061-7102], and National Bioethics Committee [515/20].

### Research team

This qualitative study was conducted to evaluate the FAST-M implementation by a multi-disciplinary research team with proficiency in OBGYN (DL, SI, RS, LS, AC, JC, CD, FP), and qualitative research in the area of perinatal health (RB, SI, BK, GK, PP).

### Sample and recruitment

HCPs, including doctors, nurses, and healthcare administrators, who were involved in the implementation of the FAST-M intervention were purposively recruited and were invited by a study team member to take part in the focus group discussion to provide their views and feedback about the intervention and its implementation outcomes. Participants were eligible to participate if they were working in areas/departments of the hospital that implemented the FAST-M intervention such as OBGYN, emergency, internal medicine, and intensive care units. Discussions were scheduled at the study site and were audio-recorded following written consent from study participants [S2 File].

Three separate FGDs were conducted with groups of 1) junior doctors; 2) nurses; and 3) senior faculties and administrators, who were involved in the process of implementation. We conducted three FGDs during phase 2 based on our observations and experiences gained from the qualitative study conducted during phase 1(adaptation) [17].

In phase 1, all healthcare providers were invited to participate from different disciplines and levels in a single group discussion. However, the combined FGD impeded the views of junior doctors and nurses due to hesitancy and reverence from senior doctors, which resulted in limited feedback. Therefore, in phase 2 we conducted separate group discussions which provided an opportunity to gain more insights and their views on implementation and explore observational results in more depth.

There were 6 to 10 participants in each FGD to have equal representation from each group. Data were collected until the point of saturation was achieved in each discussion [19], which ensured that no new findings arose from the discussions, thereby allowing participants, who indicated an interest in the opportunity, to share their thoughts and opinions openly.

### Data collection

FGDs were conducted and moderated in a local language (Urdu) by qualitative researchers and study team members (RB, SI, BK). A semi-structured interview guide was developed by the research team [S3 File] and reflected the five major domains of the Consolidated Framework for Implementation Research (CFIR) (Table 1) [20], to evaluate the outcomes of FAST-M implementation and understand participants views on its utility.

**Table 1:** CFIR domains and associated constructs.

Domains	Constructs
**One: Intervention Characteristic**	Intervention Source
	Evidence Strength and quality
	Relative Advantage
	Adaptability
	Trialability
	Complexity
	Design Quality and packaging
	Cost
**Two: Outer Setting**	Patient Needs and Resources
	Cosmopolitanism
	Peer Pressure
	External Policies and Incentives
**Three: Inner Setting**	Structural characteristics
	Networks & Communication
	Culture
	Implementation Climate
	Tension for change
	Compatibility
	Relative priority
	Organizational incentives and rewards
	Goals and feedback
	Learning climate
	Readiness for implementation
	Leadership engagement
	Available resources
	Access to knowledge and information
**Four: Characteristics of Individuals**	Knowledge and Beliefs about the intervention
	Self-efficacy
	Individual stage of change
	Individual identification with organization
	Other personal Attributes
**Five: Process and outcomes**	Planning
	Engaging
	Opinion leaders
	Formally appointed internal implementation leaders
	Champions
	External change agents
	Executing
	Reflecting and evaluating

CFIR has been used in various studies to inform qualitative processes across a range of complex interventions because this flexible framework can be tailored to different settings across multiple contexts [21]. We, therefore, used the tailored CFIR framework to understand the process of implementation of the FAST-M intervention in an entirely novel/different setting and to evaluate its outcomes.

The interview guide was iteratively reviewed and refined through the course of the study in order to ensure that, as the breadth of the responses are gathered, we could explore depth and relevance in later FGDs. FGDs lasted between 30 and 60 minutes, were recorded with permission, and transcribed verbatim. Detailed field notes were also taken during discussions to capture the non-verbal language and participants’ cues.

### Data analysis

Qualitative data gained through FGDs were transcribed in local language (Urdu) and then translated in English language by interpreters for the purpose of analysis. Data were analysed using a thematic analysis approach and deductive analysis methods [22]. The CFIR domains were used as themes and constructs as subthemes [20]. The research team conducted multiple reflexive and iterative reviews of the transcripts and tapes to familiarize themselves with the data. The analysis began soon after the first FGD and continued until we gathered information from all the three FGDs.

An audit trail was used to document our decision-making process. Data were searched for information relating to CFIR domains and constructs. Five domains and their constructs of the CFIR framework were used to guide data coding, data analysis, and reporting of the findings. A qualitative thematic analysis approach was adopted, and codes were formulated deductively from the transcripts. CFIR domains were used as predefined themes, and the constructs were used as subthemes using NVivo™ version 10 (QSR International, Pty Ltd) software [23].

A codebook was developed based on the predefined themes. Additional codes on organizational systems and policies, and sustainability of the intervention that were not part of the constructs listed in the tailored CFIR, yet providing important information on the evaluation of the FAST-M intervention were added in the codebook under predefined themes where possible. Three qualitative researchers first reviewed the codes and associated themes individually, and then all the team members reviewed the codebook together to check for potential biases and to ensure the credibility of the findings, to reflect participants’ words and meanings. The detailed thematic map was developed after consensus of all team members.

## Results

Three separate FGDs were conducted with HCPs and administrators, including one with junior doctors, such as postgraduate trainees and residents; a second with nurses; and the third with senior faculty members, consultant obstetricians and health administrators (Tables 2–4). Participants were approached by the study team. Doctors and nurses mainly from OBGYN units and ICU agreed to take part in the group discussions as most of the patients in the study during the intervention phase were enrolled from these units and they were well-informed about the implementation outcomes Hospital administrators were also involved in a group discussion to have their experiences shared from the administration viewpoint.

**Table 2:** Focus group discussion with doctors.

FGD participants	N = 7
**Job Title**	
Residents	2
Postgraduate trainees	2
Registrars	1
Senior Registrars	2
**Working experience in facility**	
> 5 years	3
1–5 years	4
**Clinical areas**	
Obstetrics and gynecology	5
ICU	2
**Gender**	
Male	2
Female	5

**Table 3:** Focus group discussion with registered nurses.

FGD participants	N = 6
**Clinical areas**	
Obstetrics and Gynecology	5
ICU	1
**Working experience in facility**	
> 5 years	1
1–5 years	5
**Gender**	
Male	1
Female	5

**Table 4:** FGD with faculties and administrators.

FGD participants	N = 9
**Job Title**	
Obstetricians	6
Health care administrators	2
ICU director	1
**Working experience in facility**	
11 years and above	5
6–10 years	2
1–5 years	2
**Gender**	
Male	2
Female	7
**Role in the hospital**	
Administration	2
Leadership	3
Clinical practices	4

Five overarching themes were developed using the CFIR domains: (I) FAST-M intervention and its characteristics; (II) Influence of outer and inner settings; (III) HCPs perceptions for implementation of FAST-M intervention; (IV) Integration into the clinical setting; and (V) Outcomes of intervention. Table 5 demonstrates the identified themes, subthemes & participants’ quotes.

**Table 5:** Themes and subthemes.

Themes	Subthemes	Illustrative Quotes
**FAST-M intervention and its significance**	Advantages of FAST-M intervention	*“FAST-M intervention helped in identifying sepsis but also the other comorbidities”*
	Evidence, strength, and quality of intervention	*“Maternal mortality and morbidity due to sepsis have reduced”*
**Influence of outer and inner settings**	Resources for patient care	*“Antibiotics and other things are manageable but the real problem we face is lab tests”*
	Healthcare providers communication and their job roles	*“There is a lack of communication between doctors and nurses”*
	Seasonal variations and patient influx	*“We have also noticed seasonal variation*, *in hot seasons sepsis is more in comparison to winters*, *in winters we get fewer sepsis patients”*
**HCPs perceptions for implementation of FAST-M intervention**	Sustainability of intervention	*“FAST-M is the guideline for sepsis and this should be enforced for sustainability”*
	Preparedness and readiness for the implementation	“*Patients are recovering now*. *Now we are able to recognize the patient’s problem”*
**Integration into the clinical setting**	Reflection and Evaluation	“*We got a line of treatment and management plan*. *This was very effective strategy”*
	HCPs Recommendations	“*Nurses can also be clinical champions”*
**Outcomes of intervention**	Improved clinical outcomes	“*The patients’ outcomes are positive”*
	Improvement in clinical processes	“*This intervention is improving clinical outcomes”*

### FAST-M intervention and its significance

#### a. Advantages of the FAST-M intervention

HCPs attitudes towards the FAST-M implementation were positive and supportive. All HCPs shared positive perceptions of using the MEOWS chart for timely identification of not only sepsis patients, but also other high-risk patients. They acknowledged that the use of red and yellow flags as triggers on the MEOWS chart facilitated HCPs in identifying and categorizing patients as low or high risk according to given cutoff values. Due to the absence of a triage system in the facility, all the obstetrics and gynecology patients initially present to the labor room at the time of admission. The MEOWS chart served as a triage screening tool for the initial assessment and monitoring of patients and helped in possible diagnoses through complete patient assessment, as stated:

“*Using MEOWS chart*, *we did not only recognize the sepsis patients but also other comorbidities like post-partum eclampsia and those patients who were deteriorating with post-partum hemorrhage*, *so this helped in identifying directly the sepsis but also the other comorbidities on admission”*. *(Obstetrician-1)*

All HCPs were supportive of the use of the MEOWS chart in their routine practices as it helped them improve the initial patient assessments considering all important parameters. The improvement in initial assessments through MEOWS chart recordings was observed during the intervention phase. Whereas the FGD participants reported their perception of a substantial improvement in overall patient monitoring.

“*What used to be there was when it was very obvious that this is sepsis only then we used to pick up that case*, *but now it’s not like that*. *The difference you can say that if we take from 100*, *it used to be 20 so now you can say 60 by using this MEOWs chart (Obstetrician-3)*

HCPs believed that the FAST-M screening tools (i.e., MEOWS chart and the decision tool) augmented the decision-making abilities of both senior and junior clinicians. The early recognition facilitated the early management of patients.

“*Our postgraduate trainees have also started picking sepsis cases*. *Previously*, *we used to just manage those patients who were detected cases of sepsis on admission but now we have started picking the new cases using the decision tool” (ICU director)*

The FAST-M intervention served as a unified system for the patient assessment and management of both suspected and confirmed maternal sepsis patients. The HCPs were supported through the management plan and each component in the treatment bundle guided their treatment approach for sepsis patients.

“*The FAST-M treatment bundle gives details on the management plan and this has helped a lot in the treatment of patients that how much fluids are recommended; which antibiotic should be given and what tests to be done” (Postgraduate trainee-2)*

HCPs were trained to use the FAST-M bundle care tools prior to its implementation following the baseline phase which helped them in applying these tools in their existing practices. The FGD participants believed that more women with maternal sepsis received the overall FAST-M bundle components within an hour during the intervention phase in comparison to the baseline phase. This finding was confirmed by the following quotes:

“*Because we have started picking up sepsis patients on time*, *we administer fluids and antibiotics within one hour of the identification of those patients*. *The blood tests are also sent earlier now to rule out the cause of infection immediately” (Postgraduate trainee-1)*

“*Patient management time is crucial to saving their lives*. *Now we have become more vigilant with administrating fluids and antibiotics on time*. *(Obstetrician-1)*

It was noted that the training programs conducted for FAST-M implementation were beneficial in improving HCPs knowledge on the use of bundle components. ‘Fluids’ which is the first component of the treatment bundle were routinely administered in the facility to patients. However, HCPs lacked guidance on fluids administration and were reluctant to administer them to patients with co-morbidities such as cardiac disease and pre-eclampsia. The health experts and obstetricians guided instructions to HCPs on appropriate fluids administration in such patients during the training programs. This helped HCPs in proper fluids administration and its monitoring, considering the guidelines provided in the treatment tool.

“*We give fluids to all patients who are scheduled for C-section*, *those who are kept NPO*, *or if we think they are dehydrated*. *But this FAST-M has helped us with the proper timelines and measurements to give the fluids” (Obstetrician-2)*

“*The problem that we were facing initially before this intervention was that we were not clear with the idea of providing fluids to patients with cardiac disease or those who had some other complications such as pre-eclampsia but now we are clear on the dose*, *and we have set some guidelines on fluids administration*. *Now without wasting any time*, *our doctors whoever is on duty provide fluids and antibiotics to patients and send their blood tests immediately*, *and then monitor those patients frequently (Obstetrician- 2)*

‘Antibiotics’ is the second component of the treatment tool. Instruction on the recommended antibiotics with appropriate dosages was given to HCPs during FAST-M training workshops by experts. This strategy improved the correct and more frequent antibiotics administration during the intervention phase and was acknowledged by HCPs:

“*The antibiotics guidelines such as first line and second line antibiotics streamlined our practices*. *We used to give antibiotics previously as well to most of our patients*, *but this first line and second line of antibiotics provided us with a clear direction*. *Previously*, *we used to give 1g ceftriaxone BD which was not effective but as it is documented in the treatment tool to give 2g ceftriaxone as stat dose within one hour to maternal sepsis patients and now we are observing its effectiveness” (Obstetrician-3)*

To identify the source of infection, the treatment tool guides in taking the patient’s history, performing clinical examinations, and routine investigations to rule out the cause and treat it appropriately. Although the practices of clinical history taking and examination were very well performed in their existing setup, some gaps were identified in routine blood tests. This deficit was acknowledged during the training and resulted in improved testing during the intervention phase and complete patient management on the basis of the findings. This finding was also confirmed by one of the group participants:

*We didn’t use to send serum lactate but now we send for suspected sepsis case and from that*, *we get an idea that this is high-risk patient and then we monitor them accordingly” (Postgraduate trainee I*I*)*

The challenge of the delay in patient transport to high-level care was due to the shortage of beds in ICU, and the excessive documentation required at the time of transport as highlighted by the participants during the training programs. The attending health administrators agreed with the suggestion of increasing the number of high-density units within the wards to overcome delays in high-level care. They agreed on transfer of critical sepsis patients as a priority with only necessary patient information required at the time of transport to ICUs. There were considerable improvements seen in timely transportation and the frequent monitoring of maternal sepsis patients during the intervention phase as stated:

“*The patients’ transportation time has now improved; we transfer critical patients immediately to ICU and then do all the documentation*, *paper and file work” (Nurse- 2)*

“*Now we have this MEOWS chart in each patient file*, *so we frequently monitor them and record the findings and see the variations in all parameters over time” (Obstetrician-1)*

#### b. Evidence, strength, and quality of intervention

All HCPs acknowledged the intervention’s quality and strength for the implementation of evidence-based and timely management of sepsis cases. The FAST-M intervention supported the improvement of existing clinical practices and improved clinical outcomes.

“*The quality of this FAST-M is that it provides us with the actions that has to be performed according to the guided timeframes such as the patient is going in this direction then he has to get management in 1 hour if the patient has yellow flags*, *then we can wait so everything is so aligned and systematic that it improves the patient care*.*” (Postgraduate trainee-II)*

In the baseline phase, patients were assessed and monitored on their routine vitals monitoring chart but a few important parameters, such as oxygen saturation, urine output, and fetal heart rate were missed due to inadequate documentation which delayed patient management. Therefore, the importance of initial patient assessment and the significance of all the parameters to recognize early warning signs were stressed in training workshops. The HCPs were also provided hands-on training on monitoring early warning signs during FAST-M training workshops using different patient scenarios [16]. This effort improved patient monitoring during the intervention phase, as stated:

“*Now it’s easier by monitoring through MEOWS chart*, *we get an idea of how many yellow or red flags are there and in what line is that patient falling*. *So*, *this tells us how to rapidly assess and manage” (Obstetrician-2)*

The FAST-M intervention also empowered junior doctors in the decision-making process and assisted in the timely management of patients as indicated by HCPs.

“*All the junior doctors*, *let’s say 4 or 5 people on duty*, *we clearly define them that this is your patient and you have to monitor this patient and you have to record their vital signs*, *and wherever you get yellow or red trigger you have to inform*. *From this*, *it has become easier to identify and manage earlier and this is very clear that you must give bundle elements*. *Any resident doctor can easily do that*, *so the first thing is this*. *We used to give these treatment components to patients previously as well*, *but we were not giving them within one hour*. *Second*, *they are aware that this patient is sick*, *so they do the next steps themselves (Senior Registrar- I)*

### Influence of inner and outer setting

#### a. Resources for patient care

Almost all HCPs indicated the shortage of nurses in the facility that serves as a major barrier to patient care. The dearth of nurses increases the workload of doctors and compromises the quality of care provided to patients. This challenge was one of the major findings during the adaptation phase and, to overcome this barrier, the FAST-M training programs provided teachings on task-sharing between doctors and nurses. The sharing and distribution of clinical responsibilities were therefore employed during the intervention phase.

“Our doctors monitor vitals such as oxygen saturation, and BP themselves. We do medications for all patients. We look after 20 patients, the admitted patients, this is the main problem. But our doctors help us with monitoring vital signs and all other tasks. (Nurse -1)

The treatment bundle components, such as fluids and antibiotics, were readily available in the facility as confirmed by HCPs, but for the component of source identification and treatment, there remained challenges in laboratory investigations, such as blood culture tests. HCPs expressed that the laboratory lacked the ability to perform blood cultures for anaerobic organisms and retained products of conception (RPOCs) which hindered decision-making on the patient’s treatment regime. However, improvement was still seen in the blood cultures in suspected sepsis patients during the intervention phase.

“*There are no cultures for anaerobes and RPOCs if that would be available it would have helped us a lot” (Senior Registrar -I)*

“*We have started sending the blood cultures for sepsis patients now*, *but still the mechanism of sending those to the lab is problematic” (Obstetrician- 2)*

#### b. Healthcare providers communication and job roles

Almost all HCPs identified lack of communication as a major barrier to FAST-M implementation. This finding was also identified during the adaptation phase and was addressed in training workshops to strengthen interprofessional communication. The communication strategies were discussed through interactive sessions and the probable solutions were identified for improvement. However, this challenge is associated with the organizational culture and will take time to change.

“*There is a lack of communication*, *it’s improving but still requires improvement*. *As we are working in wards*, *our communication with our colleagues*, *doctors*, *postgraduate trainees*, *and administrators was very poor*, *few systems have improved*, *and few have to improve but this needs to be communicated and needs more improvement” (Nurse 3)*

Moreover, a lack of trust by doctors in nurses to perform patient assessments and monitoring was expressed during the discussion. Doctors were not content with nurses performing routine tasks which caused overburdening of doctors’ responsibilities.

“*Monitoring is the responsibility of staff nurses; this is their work*, *but we can’t rely on them*, *and we can’t trust them*. *If they monitor anyone’s blood pressure*, *many a time this has happened that they record something on a chart and when we monitored*, *we noticed that diastolic BP is very high*, *how can we rely on it*? *Similarly*, *if we count heart rate it is 102 and if they do it records 72” (Postgraduate trainee-1)*

On the contrary, nurses believed that they are not responsible for patient monitoring, and this is only ‘the doctors’ duty because there were more doctors than nurses available in the facility.

“*Doctors do these duties on their wish because there are 3 doctors assigned to a single bed*, *one registrar*, *one postgraduate trainee and a house officer they do every recording themselves” (Nurse-5)*

#### c. Seasonal variation and patient influx

The effects of seasonal variation on patient influx were evident through the observational findings recorded during the baseline and intervention phases. The baseline data were collected from June to August (summer in Pakistan) and the patient admission records were 5500 patients in all OBGYN units of the study setting per month. The intervention phase was implemented from August to December (autumn and winter) and the admission records 3200 patients admitted to OBGYN units per month.

The HCPs, who worked as clinical champions, shared their views on variation in patient influx according to seasonal differences. They believed that the patient volume during the summer season was high in comparison to the winter season and this might be the reason for receiving more septic patients during summers. This could be due to difficulties in the accessibility of rural women to the city area in winters as most of the patients who visit LUMHS hospital are from rural areas. These important insights can add to interpretation of our observational results as the intervention period for this study was mostly spread over winter months.

“*We have also noticed seasonal variation*, *in hot seasons sepsis is more in comparison to winters*, *in winters we get fewer sepsis patients” (Obstetrician- 2)*

“*We get less flow of patients in the winter season*, *and this is the reason we have less rate of sepsis patients” (Resident doctor-1)*

### HCP perceptions for implementation of FAST-M intervention

#### a. Sustainability of intervention

All HCPs provided positive views regarding the long-term sustainability of FAST-M intervention. The components of the FAST-M intervention were already used in the setting; however, the intervention provided a systematic approach for patient care. Hence, HCPs were optimistic about the long-term sustainability of this intervention.

“*Every implementation requires a protocol*. *We know that we have to do NVD then we have to maintain the partograph*. *Similarly*, *this is the guideline for sepsis*, *and this should be enforced that this is a sepsis bundle*. *We are doing this for the first time*, *but these are guidelines for sepsis*, *which we have to implement accordingly*. *As we must follow partogram during delivery and no matter how busy we are we know we have to follow that so similarly this should be applied for sepsis patients and should be attached with their files*. *Every individual should know that they need to manage sepsis patients this way*. *They will remember these and will do it on tips like other guidelines” (Postgraduate trainee-2)*

The participants indicated the intent of a long-term uptake and implementation of this intervention in order to enhance future care practices and potential adoption as the protocol for all sepsis patients.

“*This is improving outcomes and then definitely this will be standard protocol in the later years and will be part of patient management in entire hospital” (HOD-OBGYN)*

The clinicians and health administrators supported the idea of long-term sustainability, and they guaranteed the inclusion of bundle care tools in patients files. As some of the practitioners working at the study setting were also practicing at other private healthcare settings at other facilities, it was suggested by the administrators that this might help in disseminating the awareness of this useful intervention in the care of patients.

“*Our doctors are running their private clinics*. *They do NVDs and c-sections at other facilities too*. *Many postgraduate trainees who are trained here will go and will serve in big reputed hospitals*. *I believe if we have trained them on this*, *we have completed the major step of dissemination already” (Healthcare administrator)*

#### b. Preparedness and readiness for implementation

As part of this study, HCPs from all participating units were trained before the actual implementation of the intervention. The study team conducted multiple-day training sessions as planned and described in the protocol to provide awareness and knowledge about the intervention [17]. However, due to the rotational duties of HCPs, all of them were not able to attend the sessions. The HCPs, who did not attend the training sessions, were not aware of the intervention and they were later trained by the clinical champions selected from their units.

“*The champions you selected trained everyone and not only postgraduate trainees*. *All HCPs were taught on FAST-M bundle care tools” (Registrar- I)*

The focus group participants expressed readiness for long term implementation of the FAST-M intervention in their setting as it improved patient outcomes and provided them with guidelines for patient care.

“*These all things were previously as well but they were not done stepwise*. *We used to do vital signs but there was no concept of red and yellow triggers*, *so we were unaware of what is going on but now on the basis of these charts*, *even in ICU*, *we use the MEOWS chart to see patients’ conditions” (Nurse-5)*

“*Patients are recovering now*. *Now we can recognize the patient’s problem*. *Previously we were unable to identify what is their problem but now they are given proper treatment” (Postgraduate trainee-2)*

### Integration into the clinical setting

#### a. Reflection and evaluation

The FAST-M intervention was very well integrated into the study setting and all participants provided positive views about the process of implementation. The role of champions was highlighted as they played a lead role in several key activities (i.e., disseminating knowledge, advocating, arranging meetings with stakeholders, tracking quality indicators, and developing organizational communication strategies and relationships).

“*The champions who were selected used to teach and train everyone and used to promote this*. *They used to check those tools in files and we also made sure that these tools are available in files*. *We were very much involved*, *and we also benefited from this thing*, *not only sepsis but there are many other high-risk patients who are picked and managed on time*. *We got a line of treatment and a management plan*. *This was a very effective strategy” (Resident doctor-2)*

The MEOWS chart assisted HCPs to assess patient’s health conditions and decide on their management and frequency of monitoring based on improvement or deterioration of clinical parameters listed and their related cutoff values. Based on the beneficial outcomes of the inclusion of the MEOWS chart, the hospital administration decided to include the MEOWS chart in patient files as their routine practice, as stated:

“*The MEOWS chart has helped a lot in improving patients’ outcomes and the hospital administration has accepted our request to include these charts in every patient’s file with other routine documents*. *These charts will be included from the time patient will be admitted to the hospital so this will help us in doing their initial assessments and frequent monitoring later” (HOD- OBGYN)*

FAST-M tools (i.e., MEOWS chart, decision, and treatment tool) were also displayed through posters in all the units involved in the management of maternal sepsis and implementation of the FAST-M intervention. HCPs supported displaying posters stating these visuals improved awareness, knowledge, and dissemination of sepsis management.

“*Sometimes by looking at these posters we get a clue of patients conditions and discuss among ourselves even if we are not assigned on that patient” (Nurse-4)*

#### b. HCP recommendations

During the process of implementation, HCPs identified certain difficulties, such as lack of HCPs awareness and training, and lack of nurses’ empowerment. They provided their views on reducing these gaps to strengthen the quality of patient care and the long-term sustainability of the intervention. The senior obstetricians advised improving awareness of HCPs and especially nurses as they could play a major part in patient monitoring.

“*If there would be refreshers and training held after every two months then this would work more effectively” (Obstetrician-4)*

“*We need improvement and I suggest providing awareness sessions to nurses especially as they could initiate the monitoring whenever doctors are busy or unavailable” (HOD-OBGYN)*

During the training workshops, HCPs selected as clinical champions showed their willingness for voluntary involvement in the FAST-M implementation. All HCPs who showed commitment to becoming champions were doctors. Therefore, the FGD participants recommended empowering nurses by providing them with a primary role in operationalizing this intervention. One of the participants suggested selecting a champion or a team leader from nurses, so they could recognize and accept their role and feel empowered to engage in these efforts.

“*As you have selected FAST-M champions for doctors similarly nurses can be made champions and this way they will be made accountable for their performance” (HOD-OBGYN)*

### Outcomes of intervention

#### a. Improved clinical outcomes

All focus group participants discussed about improved patient outcomes through the use of the FAST-M intervention. A decline in maternal mortality due to sepsis was observed after implementation of the FAST-M intervention. This was an unexpected finding as the study was not powered for such an important outcome. Interestingly, this finding was though also confirmed to be consistent with the perceptions of HCPs:

“*Maternal mortality due to sepsis have reduced because of intervening this IV fluid and antibiotics within an hour*, *the patients’ outcome is positive” (Health administrator 1)*

“*Maternal mortality and morbidity in our unit due to sepsis have reduced because of timely administration of fluids and antibiotics in an hour” (Resident doctor I)*

However, the finding can only indicate a trend, and this needs to be verified in a larger sample. Even though, the intervention not only benefited patients who were diagnosed with sepsis, but it helped improve overall patient care.

“*Now a greater number of patients are diagnosed who may remain undiagnosed*, *now only one sign we get*, *and we manage them*. *It’s a red eye-opener for us*. *As immediately we get the red signal that this patient has got one red sign in MEOWS then we manage that patient” (Postgraduate trainee-2)*

#### b. Improvement in clinical practices

The HCPs increased compliance with timely approach to patient monitoring showed significant difference during the baseline and the intervention phase in monitoring and recording of parameters such as fetal heart rate in pregnant women, urine output and oxygen saturations. The intervention improved patient monitoring as indicated by HCPs.

“*Now we monitor every patient using this MEOWS chart even if she is not a sepsis patient*. *This help us in monitoring patients’ conditions for the time they are admitted in our wards” (Postgraduate trainee-II)*

The HCPs also verbalised on the significant improvement in considering the investigational profile of patients to decide on the appropriate treatment regime. The significant increase in laboratory investigations supported clinicians in making timely decisions, as reported:

“*One of the important pieces of evidence to start any therapy or treatment is to know patients’ labs*. *This FAST-M intervention has helped us in improving lab investigations that are required for patients’ treatments (Resident doctor-II)*

The healthcare administrators were enthusiastic on inclusion of FAST-M bundle care tools in their existing practices as they observed the overall improvement in clinical practices not only of the sepsis patients but with other maternal issues. One of the administrators stated:

“*The amount of improvement these tools have demonstrated is significant*. *We have got a lot of patients with infections due to home births*, *untrained skilled workers*, *poverty*, *negligence of healthcare and many other factors*. *This will provide us a way to improve our practice and I will really urge to include these in our patients’ files so they don’t get delayed in getting care” (Healthcare Administrator)*

The FAST-M intervention improved in early identification of not only sepsis but other complicated patients, and guided on the management of patients. This helped all the healthcare workers working if different setting to work on a standard protocol. The people working in HDUs, and ICUs were also benefited through the use of these bundle care tools as stated:

“*We have a different protocol for managing sepsis patients*. *We get patients with severe sepsis so we have to start inotropes and steroid therapy when they are in late stage but yes*, *the administration of fluids*, *antibiotics and source control and continuous monitoring is even required whether if patients is in early or late stage of sepsis*, *and so it has helped us to keep a strict eye on monitoring of patients so we can manage them accordingly*.*” (ICU director)*

## Discussion

Our findings revealed that the FGDs participants views on the FAST-M intervention were positive, and they believed that the FAST-M intervention supported improving patients’ clinical outcomes.

The MEOWS chart was used as a triage screening tool for patients’ initial assessments during the intervention phase. A considerable improvement was seen in the monitoring of vital signs through the observational records, yet HCPs and administrators provided their insights, and they found the MEOWS chart as a useful tool for patient screening and predicting maternal morbidity. A similar finding was reported in a study conducted in a tertiary public hospital in Malawi. The HCPs and managers found a locally adapted early warning screening tool as an enabler to routine screening for infectious morbidities and triaging the sickest patients for priority treatment [24].

Prior to the implementation of this intervention, the patients’ vital signs were manually recorded in their files. However, the MEOWS chart was introduced in the units where FAST-M intervention was implemented and proved to improve clinical practices. Hence, the hospital administration decided to include MEOWS charts in patients’ files in all facilities for monitoring maternal patients.

The decision tool aided in the decision-making of patients who had signs and symptoms of sepsis and were detected through the MEOWS chart. The use of the FAST-M decision tool supported the clinicians to identify suspected maternal sepsis cases and directed them to make more accurate patient diagnoses. Similar findings were seen when the FAST-M intervention was implemented in Malawi. The FAST-M decision tool helped clinical decision-makers to differentiate maternal sepsis cases from other complications thereby facilitating earlier and appropriate patient management [12].

The HCPs were supportive of the treatment bundle components as they helped improve patients’ clinical conditions through timely administration. The clinical practices, such as fluid and antibiotics administration, source identification and treatment, transport, and monitoring, were already part of the existing care setup, but the FAST-M intervention provided guidance on the recommendations and guidelines for use of each component. The appropriate use of each bundle component within a recommended timeframe was found to be effective in maternal sepsis management. Similar findings were recorded when the FAST-M bundle was implemented in Malawi [12].

The FAST-M training programs helped HCPs in appropriate patient monitoring and their management. HCPs provided constructive views on the FAST-M training programs as it helped them streamline their clinical processes and discover strategies to improve the quality of patient care. The hands-on training on FAST-M tools helped them in understanding the appropriate actions that need to be followed in different situations. The training programs enabled HCPs to achieve significant improvement through their problem-solving strategies such as task sharing and strengthening the transport system of the critical patients as evidenced by their feedback obtained from group discussions.

The HCPs provided views on the challenges that were faced during the implementation such as shortage of nurses, lack of interprofessional communication, challenges with transport of blood samples, and complex transport mechanism of patients to higher level of care. However, similar challenges were reported during the adaptation phase. Yet, healthcare administrators provided their contribution in facilitating the transport mechanism of patients during the intervention phase. There were more HDUs built in within the OBGYN units to ease the complex process of patient’s transport to high level of care. Though, we believe that the long-term implementation of these strategies depends upon the organizational structure, preference of people working there and the institutional culture. Research evidence suggests that bringing innovation into the organization is a complex and time taking process and depends on the organization’s structure and its problem-solving strategies [25].

The involvement of healthcare administrators and key stakeholders in the adaptation phase also helped in instilling and implementing the idea of task sharing during the intervention phase. Task sharing has been a longstanding strategy in health systems and health settings and has proved to improve clinical practices and outcomes [26]. The approach of task sharing of specialists with trained non-specialist workers has provided positive outcomes in the improvement of patient care, reduced morbidity and mortality rates, and cost-effectiveness [27].

During phase 1, it was proposed to involve nursing interns, trainee dispensers and other available human resources to reduce doctors and nurses’ workload [17]. Since the intervention was implemented during the period when the COVID-19 pandemic was at its peak, most of them got involved in additional services such as telemedicine, vaccinations, and data collection of infected patients (COVID-19). Therefore, the idea of involving other human resources could not be achieved during the intervention phase. However, during evaluation phase, the participants continued to encourage the idea of task sharing and were enthusiastic to implement this model in their health system as a long-term work.

The participants acknowledged the benefits of co-interventions, such as the selection of clinical champions and posters demonstrating the FAST-M tools played an important part in the dissemination of knowledge about the FAST-M intervention. The active role of champions improved the patients and clinical outcomes through continuous monitoring and ongoing evaluations. Literature provides positive accounts of monitoring and evaluation, especially in healthcare developments, such as the development of a novel health information project in a health setting [28]. As the FAST-M intervention is implemented for the first time in an entirely new setting, continuous monitoring and evaluations by the senior trained healthcare professionals are necessary to support its establishment and normalisation into practice so it is sustainable in the long term.

The HCPs and administrators also supported sustainability and long-term implementation of the FAST-M intervention. HCPs believed that the healthcare administrator’s role could further strengthen the use of the bundle approach. They also empowered healthcare providers working in other facilities to uptake this approach and further disseminate awareness of its use.

The major strength of this study is the use of CFIR which guided the researchers’ focus and provided a predefined framework for the analysis of findings. The CFIR framework helped us in reflecting the five major domains [21] to evaluate the study outcomes and understand HCPs’ views on the effectiveness of FAST-M intervention. The CFIR framework also guided us in understanding the processes required for its implementation in an entirely new setting. Moreover, three separate focus group discussions with different groups of HCPs and health administrators that helped us in gaining their views and experiences to have better insights and knowledge regarding the process of implementation from each level.

The study also has some limitations. First, the study focused only on the perspectives of HCPs and administrators who were involved in the implementation of the FAST-M intervention. The focus on their perspectives might have let us miss the perspectives of patients and their families. Lastly, the intervention was implemented in only one study setting in Pakistan at this time. However, it is noted that this site serves a diverse population from the urban and rural areas of the province of Sindh.

We believe that the FAST-M intervention is feasible for use in the low-resource settings of Pakistan, and we recommend several strategies to address the challenges that were encountered during the implementation. The organisational leadership and HCPs require collaboration to work as a multidisciplinary team. The organizational structure and culture require modifications to strengthen HCPs linkages and relationships, which may be catalyzed through the empowerment of nurses and junior doctors who are important in the continuum of care.

The distribution of supportive resources to provide continuous education to doctors, nurses, and healthcare administrators about sepsis care is required to increase knowledge and awareness of sepsis care approaches. Also, facilities will require selected team leaders for the ongoing monitoring and evaluation of health indicators.

We have identified the feasibility of this intervention in one facility in Pakistan selected as our study site. The FAST-M tools had been previously adapted to ensure they were appropriate for the existing sepsis practices and circumstances of the current study setting. These findings show that this adaptation had been successful. If the FAST-M intervention is going to continue to expand to be used in other contrasting countries, we would recommend that a similar adaptation process should again be undertaken to ensure it is optimized for the new context. Future research is also needed to understand the feasibility of this intervention at the primary and secondary levels of care to reduce the delays in patient care for those patients who are referred from these facilities.

## Conclusion

The FAST-M intervention has the potential to be used as an integrated approach for early recognition and management of maternal sepsis in low-resource health settings in Pakistan. The HCPs and health administrators’ views and experiences from this study were encouraging in the implementation of the intervention and its relative outcomes.

This paper provided qualitative findings on the outcomes of the FAST-M intervention and provided us with rich data to explore clinical outcomes and provided important information to support subsequent refinement and scaling of the intervention.

## Supporting information

S1 Checklist ISSM-COREQ-checklist. (PDF)

S1 File FAST-M bundle care tools. (PDF)

S2 File Participant written consent form. (PDF)

S3 File Interview guide. (PDF)
