# Supplementary material for: Evaluation of the FAST-M maternal sepsis intervention in Pakistan: A qualitative exploratory study
Source: PLoS One. 2023 Apr 24;18(4):e0284530. doi: 10.1371/journal.pone.0284530 (PMC10124821; doi:10.1371/journal.pone.0284530)
Supplement: S1 File — (PDF) [file pone.0284530.s002.pdf]

MODIFIED EARLY OBSTETRIC WARNING CHART (MEOWS CHART)

FAST-M

Contact clinical decision maker if patient triggers **ONE RED** or **TWO YELLOW** flags at any one time.

Patient		Patient ID		DOB/Age	

WRITE **VALUES** IN BOXES PROVIDED

Date															
Time															
Initials															

**Respiratory rate** (breaths per minute)	25 or more	RED															
	21 - 24	YELLOW															
	11 - 20	NORMAL															
	10 or less	RED															
**Oxygen saturations** (%)	95 or more	NORMAL															
	94 or less OR needing oxygen	RED															
**Temperature** (°C)	38 or more	YELLOW															
	36.0 to 37.9	NORMAL															
	35.9 or less	YELLOW															
**Heart rate** (beats per minute)	120 or more	RED															
	100 - 119	YELLOW															
	50 - 99	NORMAL															
	40 - 49	YELLOW															
	39 or less	RED															
**Systolic blood pressure** (mmHg)	160 or more	RED															
	140 -159	YELLOW															
	100 -139	NORMAL															
	90 - 99	YELLOW															
	89 or less	RED															
**Diastolic blood pressure** (mmHg)	110 or more	RED															
	90 -109	YELLOW															
	40 - 89	NORMAL															
	39 or less	RED															
**Urine output** Hours since patient passed urine (tick box)	12 hours or less	NORMAL															
	12 - 18 hours	YELLOW															
	18 hours or more OR less than 0.5 mL/kg/hour	RED															
**Mental State** (tick box)	Alert	NORMAL															
	Not Alert	RED															
**Looks unwell** (tick box)	No	NORMAL															
	Yes	YELLOW															
**TOTAL YELLOW FLAGS**																	
**TOTAL RED FLAGS**																	
**ACTION TAKEN (IF REQUIRED) Yes (Y) / No (N)**																	

DECISION TOOL

FAST-M PATHWAY

Patient name		Staff name	
Date of birth or age		Role	
Patient ID		Signature	
Date	____/____/____	Time	_____ : _____

START HERE

- ☐ Abnormal vital signs (Resp rate / Temp / HR / BP / Urine output / Mental state) or MEOWS chart trigger

OR

- ☐ Concerned about a potential maternal infection

OR

- ☐ Fetal tachycardia (greater than or equal to 160 beats per minute)

NO → LOW RISK OF SEPSIS

YES ↓

COULD SHE HAVE AN INFECTION?

- ☐ Abdominal pain or distension
- ☐ Breast abscess / mastitis
- ☐ Chorioamnionitis / endometritis
- ☐ Infected cannula / line
- ☐ Infected perineal / abdominal wound
- ☐ Lower respiratory tract infection
- ☐ Meningitis
- ☐ Severe sore throat
- ☐ Urinary tract infection
- ☐ Yes, but source unclear
- ☐ Other (specify)

NO → LOW RISK OF SEPSIS

ANY SEPSIS RED FLAG PRESENT?

- Respiratory rate **25 breaths per minute or more**
- Oxygen saturations **94% or less OR oxygen needed to keep saturations 95% or more**
- Heart rate **120 beat per minute or more**
- **Systolic blood pressure** 89 mmHg or less
- Not passed urine **in over 18 hours/less than 0.5 ml/kg/hr if catheterized**
- Mental state **Not Alert**

NO → ANY TWO SEPSIS YELLOW FLAGS PRESENT?

YES ↓ START FAST-M BUNDLE NOW

ANY TWO SEPSIS YELLOW FLAGS PRESENT?

- ☐ Respiratory rate
  21 - 24 breaths per minute
- ☐ Temperature
  35.9°C or less OR 38°C or more
- ☐ Heart rate
  100-119 beats per minute
- ☐ Systolic blood pressure
  90 - 99 mmHg
- ☐ Passed urine in last
  12 - 18 hours
- ☐ Looks unwell
- ☐ Risk factors
  (e.g. immunosuppressed / steroids / diabetes)

NO → LOW RISK OF SEPSIS

YES ↓

REVIEW BY CLINICAL DECISION MAKER

Review by clinical decision maker within 3 hours and continue hourly maternal observations.

Review taken place within 3 hours? ☐ YES ☐ NO

Date ___/___/___ Time ____ : ____

Antibiotics required? ☐ YES ☐ NO

IF ANY RED FLAGS DEVELOP → START FAST-M BUNDLE NOW

START FAST-M BUNDLE NOW

Review by clinical decision maker and **actions within ONE HOUR**.

LOW RISK OF SEPSIS

Review and manage accordingly.
Monitor inpatients with MEOWS chart.
Educate outpatients on warning signs.

UNITED AGAINST MATERNAL SEPSIS

THINK SEPSIS, ACT FAST-M

TREATMENT BUNDLE

FAST-M

REMEMBER TO COMPLETE THESE ACTIONS WITHIN ONE HOUR

Patient name		Staff name			
D.O.B or age		Role/Cadre			
Patient ID		Signature			
Date & time of red flag observation	__/__/__ __:_	Date & time bundle started	__/__/__ __:_	Date & time of review by clinical decision maker	__/__/__ __:_

F

FLUIDS (caution in pre-eclampsia, severe anaemia and pulmonary oedema)						
Date	__/__/__	Time fluids initiated	__:__	Initials		Give 500 ml crystalloid immediately. Repeat 500 ml boluses to a maximum of 30 ml/kg if hypotension persists.
Details / reason not completed						

A

ANTIBIOTICS						
Date	__/__/__	Time started	__:__	Initials		See antibiotic guidelines below
Details / reason not completed						

S

SOURCE – identify and treat the source of infection						
Date	__/__/__	Time considered	__:__	Initials		See source identification and treatment boxes below
Details / reason not completed						

T

TRANSPORT (to higher level hospital or location within hospital, if required)					
Date & time transport considered	__/__/__ __:__	Initials		Transport Required	☐ YES ☐ NO
Date & time transport requested	__/__/__ __:__	Initials		☐ N/A	
Date & time patient left facility	__/__/__ __:__	Initials			
Destination					
Reason for any delay					

M

MONITORING (start MEOWS chart if not already started. Repeat observations every 30 minutes until otherwise decided by clinical decision maker)		
Date & time monitoring commenced	__/__/__ __:__	Details / reason not completed
Maternal / fetal monitoring should include	• Respiratory rate<br>• Oxygen Saturations<br>• Temperature<br>• Heart rate<br>• Blood pressure<br>• Urine output<br>• Mental state<br>• Fetal heart rate	
Neonatal monitoring and review commenced	☐ YES ☐ NO ☐ N/A	

ANTIBIOTIC GUIDELINES

Insert local guidance here

Immediate treatment for Maternal Sepsis:

- Ceftriaxone 2 g IV once daily
  (if no IV access this can be given as 2 IM injections of 1 g in different sites).
- If possible intra-abdominal source add Metronidazole 500 mg IV three times daily or 400 mg PO three times daily.

If above antibiotic regime is not available then give:

- Tazobactam 4.5 g IV daily two time a day
- Meropenem 1 g IV daily two times a day

IDENTIFY THE SOURCE

Consider

• Clinical history	• Blood cultures	• Sputum sample
• Clinical examination	• HIV and Malaria testing	• Imaging (abdominal, chest)
• Blood tests (if available) (FBC, U&Es, LFTs, CRP, clotting)	• Urine sample	• Lumbar puncture
	• Swabs (wound, vagina, throat)	• Other ____________

REMOVE / TREAT THE SOURCE

Consider

• Malaria treatment	• Removal of infected cannula / line
• Consider delivery of baby	• Hysterectomy
• Removal of retained products of conception	• Targeted antibiotics once source known
• Debridement of wound / drainage of collection	

UNITED AGAINST MATERNAL SEPSIS

THINK SEPSIS, ACT FAST-M
