# Supplementary material for: Evaluation of the FAST-M maternal sepsis intervention in Pakistan: A qualitative exploratory study
Source: PLoS One. 2023 Apr 24;18(4):e0284530. doi: 10.1371/journal.pone.0284530 (PMC10124821; doi:10.1371/journal.pone.0284530)
Supplement: S2 File — (PDF) [file pone.0284530.s003.pdf]

Informed Consent

Title of study:	Extension of the FAST-M maternal sepsis bundle in Pakistan, a feasibility study
Chief Investigator:	Professor David Lissauer
Site:	Liaquat University of Health Sciences<br>Pakistan
Site Principal Investigator:	Dr Sheikh Irfan Ahmed
Site CO-PI's	Dr Lumaan Sheikh, Dr Raheel Sikandar and Dr. Rubina Barolia
Ethics approval:	AKU ERC-2019-2061-7102,<br>LUMHS/ REC/-886, 4-87/NBC-515/20/
Affiliated organizations:	University of Birmingham, University of Liverpool & Aga Khan University Hospital Pakistan & Liaquat University of Medical & Health Science, Jamshoro.

We would like to invite you to take part in this research study. Before you decide, we would like you to understand the study, why the research is being done and what this part of the study involves for you. One of the team will explain the study to you and answer any questions you may have.

Part 1: Purpose of the study

What is the purpose of the overall study?

We are developing an intervention that we hope will improve the care of patients with maternal sepsis around the world. Sepsis is when an infection has become severe enough to lead to organ dysfunction and become life threatening.

The intervention is composed of three things:

1. The MEOWS (Maternal Early Warning Scores) chart tool to help you monitor patient's observations and help detect maternal sepsis

2. The FAST-M sepsis "bundle", to help ensure fast, consistent and effective treatment of maternal sepsis

3. A training day to learn to use the tools to help recognize and treat maternal sepsis

We hope that this intervention will make caring for patients with maternal sepsis easier. This study aims to discover whether it is possible to introduce this intervention into Pakistan healthcare facilities.

We hope to try and understand the good and bad aspects of the bundle to try and make it more user friendly and effective. We hope that using this bundle will make caring for patients with maternal sepsis easier.

In order to achieve this we hope to:

1. Understand your current experiences in managing maternal sepsis at your hospital

2. Understand what you thought was good and bad about the intervention.

3. Understand ways to improve the intervention.

4. Evaluate the intervention to see if it improves care in your hospital.

We hope you will be willing to participate in all of the activities for the study mentioned above.

Why have I been invited to participate?

You have been invited to participate because you work in maternity care and we would like to understand your experiences of maternal sepsis and the proposed intervention.

What will I have to do if I take part?

You will be interviewed several times over a period of six to eight months. Sometimes these will be one on one interviews and sometimes in groups. The interviews will be in English and take up to an hour. The interview will take place at or close-by to your place of work, at a time that is convenient to you. The interview will be audio-recorded to allow us to analyse the information you give us. Some or all of the information will be transcribed word for word. This information will be used in several ways – all of which will be anonymous so that your identity is not disclosed. The table describes how your information will be used.

At the start of the study the information that you give us will be used to understand current practice at your hospital for the management of maternal sepsis. During the study the information that you give us will be used to discover the good and bad aspects of the intervention and how it could be improved to make it easier for you to manage patients with maternal sepsis. This will help us decide whether the intervention is a success or not. Some of the information you give us, including word for word extracts, will be used in the final project report, which may also be published in a journal.

Do I have to take part?

It is completely up to you to volunteer to be interviewed and it will have no effect upon your work. We will describe the study and go through this information sheet with you. If you decide to take part, we will then ask you to sign a consent form.

What are the possible disadvantages and risks of taking part?

Before participating you should consider that we will be asking you about your experiences, opinions, beliefs and feelings in relation to the intervention. We are interested in finding out about the positive things that help you do your work and anything that hinders your work. Although unlikely, there is a possibility that you might feel upset when answering these questions during the interview. If this was to occur, you would be able to take a break or continue another day.

There will be an opportunity at the end of the interview for you to consider whether there is anything that you have discussed that you would prefer not to be included in the transcript. The transcript will also be made available to you to review by email if you would like. As a participant you are free to withdraw during the interview and up to a month afterwards, without giving a reason.

What are the possible benefits of taking part?

We hope that you will find the experience interesting and enjoyable. The information we collect from this study will be used to help us make the intervention the best it can be. Your interview will also be very important in evaluating the interventions effects at your hospital and its potential usefulness in the management of maternal sepsis.

What are the financial considerations of taking part in this study?

We would like to provide you a token of thanks at the end of the interview for providing your time and information with us.

What if there is a problem?

Any complaint about the way you have been dealt with during the study or any possible difficulty you might suffer will be addressed. Information on this is given in Part 2.

Will my taking part in the study be kept confidential?

We will follow ethical practice and all information about you will be handled in confidence. Further details are included in Part 2.

This completes part 1. If the information in Part 1 has interested you and you are considering participation, please read the additional information in Part 2 before making any decision.

Part 2: Conduct of the study

What will happen if I don't want to carry on with the study?

You may withdraw from the study without giving a reason. If you chose to withdraw from the study during or up to one month after your interview, we might ask you whether we can use the information you have given us, such as your interview answers. If you don't want to carry on with the study but you give us permission to use the information already collected, we will proceed to keep it securely. If you wish to withdraw and don't want your data to be used for the study, we will delete any recordings and destroy transcript files.

What if there is a problem?

If you have a concern about any aspect of this study, you can speak to the researchers, who will do their best to answer your questions. Their contact details are on the last page.

Will my taking part in this study be kept confidential?

The study will take place at your workplace, and for this reason it is possible that other work colleagues will be aware of your participation. However, we will follow these procedures for collecting, storing, processing and destroying information about you to ensure your confidentiality and safeguard your data:

- The recording of any information you give us during your interview will be stored in a password protected file and only authorised people will have access to it. This will help prevent people identifying your voice.
- The data transcribed from recordings will be stored securely on a computer with access restricted by a password. Transcripts will not include names or locations. Consent forms and printed transcripts will be kept in a locked cabinet, only accessible to authorised researchers.
- Data collected will be used for this study but, with your permission, might also be retained to include it anonymously in future studies.
- The identifiable data will be retained for the duration of the study and will be disposed of securely (i.e. shredding documents).

As a participant, you would have the right to check the accuracy of data held about you and correct any errors.

What will happen to the results of the research study?

The researchers will write a report outlining the results of this study. You will not be identified in any report, presentation or publication, however extracts from your interviews may be reproduced. The results will be used to inform local practice and a future possible larger scale trial of the intervention. If you are interested in the outcome of the research, then a summary of the findings can be sent to you via email and if you wish you will be invited to attend a feedback day at the end of the project.

Who is organizing the research

This study is being carried out by the University of Birmingham, UK. University of Liverpool, UK and Aga Khan University Hospital(AKUH), Pakistan The research team is being led by Dr David Lissauer, Dr Lumaan Sheikh and Dr Sheikh Irfan is the researcher conducting this part of the study.

Who has reviewed the study?

This study has been reviewed by the National Bioethics Committee Pakistan and College Research Ethics Committee in AKUH.

Contact details:

Dr Sheikh Irfan Ahmed, Senior Instructor, AKUH National stadium road, Karachi Email: sheikh.irfan@aku.edu Telephone number: +92-021-34864650

Dr David Lissauer Lecturer in Maternal and Fetal Medicine, University of Birmingham, UK Email: David.Lissauer@liverpool.ac.uk

Dr Lumaan Sheikh Associate Professor, AKUH National stadium road, Karachi Email: lumaan.sheikh@aku.edu Telephone number: +92-021-34864641

Dr Raheel Sikandar Professor, Liaquat University of Medical & Health Sciences, Jamshoro Email: pgmc@lumhs.edu.pk Telephone number: + 92-22-9213322

Please keep this information sheet for your own records.

Dr Rubina Barolia, Associate Professor and Assistant Dean, School of Nursing, AKU, Email: rubina.barolia@aku.edu Telephone number: +92-021-34865446

Bakhtawar Khowaja, Research Coordinator, AKUH National stadium road, Karachi Email: Bakhtawar.hanif@aku.edu Telephone number: +92-021-34864626

PLEASE INITIAL THE BOXES IF YOU AGREE WITH EACH SECTION:

1. I have read the information sheet version 2.5 for the above study and have been given a copy to keep. I have had the opportunity to consider the information, ask questions and have had these answered satisfactorily. ☐

2. I understand that my participation is voluntary and that I am free to withdraw up to one month after my participation without giving any reason. ☐

3. I agree to be interviewed for research in this study. I agree to my interview being audio-recorded and I understand that transcripts will be anonymised. I understand that participating in the interview for this research is voluntary and that I am free to withdraw my approval for use of the audio recordings and transcripts up to one month after my participation. ☐

4. I understand that anonymised sections of data collected during the study, may be looked at by individuals from regulatory authorities in the UK or Pakistan. I give permission for these individuals to have access to my anonymised transcript. ☐

5. I understand that the researchers might publish an article in a journal with the results of this study. I give permission for my transcripts to be used for this purpose. I understand that these transcripts will be anonymised. ☐

6. I know how to contact the research team if I need to. ☐

7. I understand that I may terminate the interview at any time ☐

8. I am happy for information about me related to the study being stored on a password protected computer system, which will be backed-up in a separate location to keep this information safe. Data collected will be used for this study but, might also be retained to include it anonymously in future studies ☐

9. I agree to participate in this study. ☐

SIGNATURES:

Participant Name and Surname ___________________ Date________________

Signature __________________________

Researcher Name and Surname _________________________ Date ___________________

Signature______________________________
