# Supplementary material for: Evaluation of the FAST-M maternal sepsis intervention in Pakistan: A qualitative exploratory study
Source: PLoS One. 2023 Apr 24;18(4):e0284530. doi: 10.1371/journal.pone.0284530 (PMC10124821; doi:10.1371/journal.pone.0284530)
Supplement: S3 File — (PDF) [file pone.0284530.s004.pdf]

Study guide for focus group (Evaluation of implementation)

Thank you so much for reading the participant information sheet and consenting to participate in our research project.

Questions:

Let's start the discussion by talking about the FAST-M intervention and its implementation in your study setting

1. How different was this intervention from your existing practices and how it was integrated into your hospital setting?
Probes: charts, communication, systematized plan for monitoring, patient documentation, records

2. Did you have sufficient resources to implement the intervention?
Probes: Fluids, antibiotics, laboratory, ambulances, stretchers, FAST-M tools, monitoring equipment, human resource

3. Who were the key individuals who helped in the implementation of the FAST-M intervention?
Probes: doctors, interns, Hos, nurses, residents, admin, HODs

4. What are your views about using the MEOWs chart for early detection of maternal sepsis cases?
Probes: red triggers, yellow triggers, pre-eclamptic cases, patients condition, frequent monitoring

5. How effective were the decision tool and treatment bundle in the timely management of sepsis patients? Overall bundle applicability of Fluids, Antibiotics, Source control, Transport, and monitoring for management of maternal sepsis cases?

Probes: clinical review, timely administration of treatment bundle, timely administration, need for administration, reasons for not administring, availability of resources

7. Do you think the intervention was effective in your setting? What were the facilitators and barriers to this intervention?
Probes: resources, the rigidity of HCPs to change the existing policies, compliance, administrative and clinical support, the display of posters, clinical champions, refreshers, training, monitoring visits, coordination with HODs, Patient improvement, length of stay of patients, patient transfer

8. Do you think this intervention helped to improve maternal and newborn health outcomes?
Probes: length of stay, fetal and neonatal wellbeing, transfer of newborn to pediatric critical units, condition of mothers and newborns at discharge, follow-up status of patients

9. What are your views about the sustainability of this intervention in your setting?
Probes: sepsis policies, protocols, integration of FAST-M intervention

That concludes our focus group. Thank you so much for coming and sharing your thoughts and opinions with us. We have a short evaluation form that we would like you to fill out if you have time. If you have additional information that you did not get to say in the focus group, please feel free to write it on this evaluation form.
